# Spontaneous and deliberate future thinking: a dual process account

**DOI:** 10.1007/s00426-019-01262-7

**Published:** 2019-12-05

**Authors:** Scott Cole, Lia Kvavilashvili

**Affiliations:** 1 School of Psychological and Social Sciences, York St John University, York, YO31 7EX UK; 2 University of Hertfordshire, Hatfield, UK

## Abstract

In this article, we address an apparent paradox in the literature on mental time travel and mind-wandering: How is it possible that future thinking is both constructive, yet often experienced as occurring spontaneously? We identify and describe two ‘routes’ whereby episodic future thoughts are brought to consciousness, with each of the ‘routes’ being associated with separable cognitive processes and functions. Voluntary future thinking relies on controlled, deliberate and slow cognitive processing. The other, termed involuntary or spontaneous future thinking, relies on automatic processes that allows ‘fully-fledged’ episodic future thoughts to freely come to mind, often triggered by internal or external cues. To unravel the paradox, we propose that the majority of spontaneous future thoughts are ‘pre-made’ (i.e., each spontaneous future thought is a re-iteration of a previously constructed future event), and therefore based on simple, well-understood, memory processes. We also propose that the pre-made hypothesis explains why spontaneous future thoughts occur rapidly, are similar to involuntary memories, and predominantly about upcoming tasks and goals. We also raise the possibility that spontaneous future thinking is the default mode of imagining the future. This dual process approach complements and extends standard theoretical approaches that emphasise constructive simulation, and outlines novel opportunities for researchers examining voluntary and spontaneous forms of future thinking.

Episodic future thinking is the ability to imagine or simulate events that may or may not occur in the future (Atance & O’Neill, 2001). It allows humans to engage in complex acts of future-oriented behaviour (Tulving, 2005), and has now garnered substantial interest from researchers of cognition, neuropsychology, and neuroscience (for a recent review, see Schacter, Benoit & Szpunar, 2017). In this paper, we focus on, and address, a central assumption of research on future thinking that has dominated the field over the past decade, namely that future thinking is a constructive and effortful process (see Schacter, 2012; Buckner & Carroll, 2007; Hassabis & Maguire, 2007; Suddendorf & Corballis, 2007; Wheeler, Stuss & Tulving, 1997). Despite this focus on constructive processes, recent findings from related fields of involuntary mental time travel and mind wandering have provided undeniable evidence that future thoughts can be experienced with the same phenomenological ‘richness’ as those elicited constructively, but without effort and conscious intent. Here, we refer to such mental experiences as involuntary or spontaneous future thoughts (Bernsten, 2019; Cole & Kvavilashvili, 2019, for definitions), and pose the following question: If future thinking is predominantly constructive, how is it possible it is often experienced as occurring spontaneously?

In this position paper, we put forward a dual process account of future thinking. Within this account, we question the view that episodic future thought typically involves more executive processes than remembering (see Schacter, Addis, Hassabis, Martin, Spreng, & Szpunar, 2012 for a review). Instead, we propose that episodic future thinking can occur via two ‘routes’ which are associated with separable cognitive processes: (a) a slow, voluntary route that involves wilfully constructing and elaborating a scenario and (b) a rapid, spontaneous route that often involves reincarnating a ‘pre-made’ future scenario. It is proposed that voluntary and spontaneous future thinking are related in a sequential fashion—(a) → (b)—such that the majority of spontaneous future thoughts can be traced back to their original constructed ‘event’ (e.g., (a)^1^ → (b)^1^; (a)^2^ → (b)^2^; (a)^3^ → (b)^3^…). We believe this approach is the most parsimonious way of interpreting findings from research on voluntary future thinking, involuntary future thinking, prospective memory and mind wandering, and reflects more accurately how humans imagine the future in everyday life (cf. Kvavilashvili & Rummel, 2019).

## The standard approach to episodic future thinking

The modus operandi of laboratory-based psychological research, especially in cognitive psychology, has been to present participants with stimuli (which can be manipulated as independent variables) and ask for responses (representing the dependent variable/s). Research on future thinking has largely followed this template: almost all future thinking studies have used standardised instructions which specify the type of event that participants must simulate (e.g., a future event related to a cue-word ‘park’/‘5 years in the future’; see Addis, Wong & Schacter, 2008; Cole, Morrison & Conway, 2013; D’Argembeau & Van der Linden, 2004), and researchers measure responses via subjective rating scales (e.g., vividness, see D’Argembeau & Van der Linden, 2004) or by coding the verbal output (episodic detail, see Addis et al., 2008). This paradigm for studying future thinking has relied heavily on the cue word method used in the study of autobiographical memory where participants are asked to interrogate their autobiographical memory knowledge base to recall a specific event in response to a given cue word (Crovitz & Shiffman, 1974; see Conway, 2005; Conway, Justice & D’Argembeau, 2018, for reviews). However, a unique configuration of task instructions is employed in studies of future thinking by requesting that imagined future events are specific and “plausible, given the participant’s plans, and novel, that is, not previously experienced by the participant” (p. 35, Addis et al., 2008; see also Cole et al., 2013; D’Argembeau & Van der Linden, 2004).

In our view, the fact that episodic future thinking has been investigated using paradigms that favour deliberate constructive processes, and that have a series of ‘rules’ not present in studies of autobiographical remembering, naturally contributes to the evidence from cognitive neuroscience (e.g., Addis, Wong & Schacter, 2007; Okuda et al., 2003) and neuropsychology (deVito, Gamboz, Brandimonte, Barone, Amboni & Della Sala, 2012) that future thinking, in general, is associated with cognitive control processes, and that these are greater than those required in autobiographical remembering. For example, the well-established constructive episodic simulation hypothesis delineates how both episodic memory and future thinking use the same re/constructive processes, but also how episodic future thoughts can be simulated by additional extraction and integration of autobiographical details (i.e., people, places and objects) from long term memory to arrive at a novel representation of a future event (Schacter & Addis, 2007). Undoubtedly, implementation of standardised instructions across studies has led to a set of convergent and reliable findings and principles, which has been widely beneficial to the field, leading to its popularity and many breakthroughs (see Klein, 2013; Schacter et al., 2012 for reviews). However, the use of certain paradigms which are ideally designed and suited to elicit deliberate constructive processes (what we term ‘the standard approach’) can give rise to the idea that future thinking is by its nature, a constructive process (e.g., Hassabis & Maguire, 2007; Schacter & Addis, 2007; Suddendorf & Corballis, 2007).

Other influential theoretical approaches to future thinking have similarly elaborated on the controlled processes involved in constructing a future scenario, and were largely based on evidence from event construction studies (Buckner & Carroll,^2007^; Hassabis & Maguire, 2007; Suddendorf & Corballis, 2007). Within these theories, the central question primarily concerned the best ways of measuring and explaining constructive processes in future thinking, without considering the possible role/s of spontaneous cognition in future thinking.

**Fig. 1:**
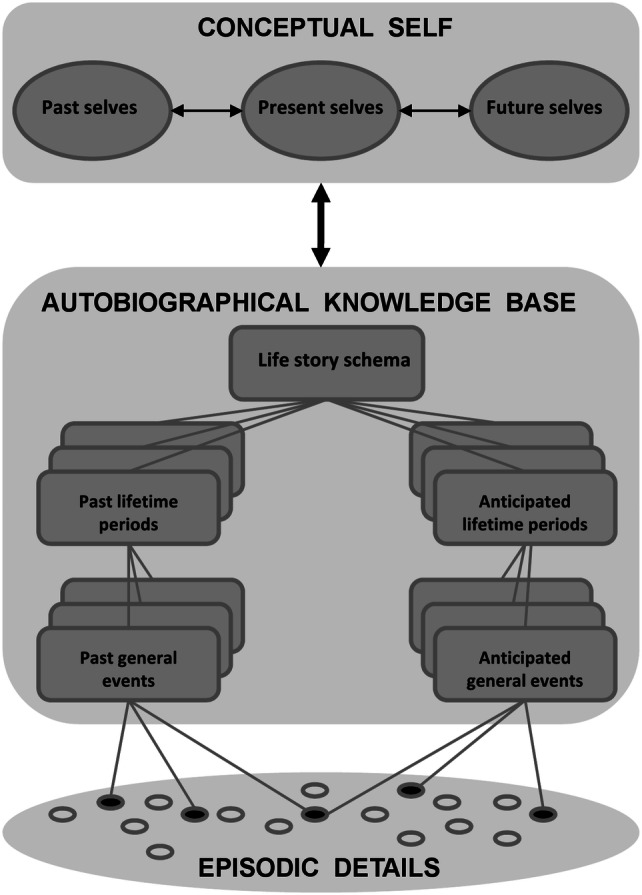
Knowledge structure representing autobiographical memory and future thought in the revised Self memory system (Conway et al. 2019) (Image taken from Conway et al. (2019). With permission from Martin Conway)

These approaches may have been influenced, in part, by traditional approaches to voluntary memory retrieval, the ‘flip-side’ of episodic future thinking (Schacter & Addis, 2007), which invoked controlled recollection or strategic retrieval as its core process to mentally revisit the past (see Conway, 2005; Tulving, 2002 for reviews). Controlled recollection is thought to involve iterative sequences of memory searches (typically from self-concept information through general lifetime information to more specific event-based information), in which a specific event is eventually constructed based on the goals of the specific search (Conway, 2005). An analogous process is thought to occur in episodic future thinking (Conway, Justice & D’Argembeau, 2019 (for more details see the section below on ‘Related theoretical models’ and Fig. 1 for a representation of this model).

Furthermore, in Conway et al.'s, updated autobiographical memory system, there are two parallel and hierarchically organised autobiographical knowledge structures, referring to ones’ past and one’s future, respectively, which can both vary in terms of specificity of mental representations at different levels of the hierarchy (Conway et al., 2019). Here, we describe the various ‘levels’ of future-based knowledge specified within this model, which interact to enable humans to imagine specific episodic future events (Atance & O’Neill, 2001; Szpunar, 2010). The most abstract is the conceptual self which represents knowledge of future self-images, such as ‘I will be a mother’ or ‘I will be a professor’ (Rathbone, Conway & Moulin, 2011). Of intermediate specificity is the level corresponding to one’s life story (McAdams, 2001) and lifetime periods in which future periods are represented, such as ‘When I have a family’ or ‘when I am retired’ (see Thomsen, 2015). More precisely constrained are general events, representing event structures or scripts common to that lifetime period, for example ‘Taking my child to school in the mornings’ or ‘playing golf on Sundays’ (Anderson & Dewhurst, 2009). Most specific are episodic representations, or episodic future thoughts, which uniquely enable one to form mental representations using episodic-specific knowledge, such as people, setting and objects (Addis et al., 2008; D’Argembeau & Van der Linden, 2004). Importantly, abstract levels constrain the content at more specific levels, thus, abstract autobiographical knowledge has a role in determining the content of episodic future thoughts (Conway et al., 2019), as can be observed when the iterative process of event construction is inspected via the use of think aloud tasks (D’Argembeau & Mathy, 2011).

However, these theories do not explicitly offer an account of the role of spontaneous processes in future thinking (but see Jeunehomme & D’Argembeau, 2016). What of future thoughts that arise in consciousness ‘out of the blue’ (i.e., unintended) and do not appear to require constructive processes?

## Unresolved questions in the standard approach

Our view is that there is now a substantial body of evidence that questions the generality of the standard approach to episodic future thinking and suggests that, in everyday life, future thoughts occur via two related, but largely independent, cognitive processes. We outline key pieces of evidence below.

Firstly, it is not always found that episodic future thinking is more effortful than episodic remembering. In several studies, when explicitly testing this idea, a clear difference between executive processing in past and future thinking was not evidenced (Anderson, Dewhurst & Nash, 2012; Cole et al., 2013; Rasmussen & Berntsen, 2018) and latency to generate an event—often a proxy measure of effort—has failed to distinguish past and future events in landmark studies in the field (Addis et al., 2007; D’Argembeau & Van der Linden, 2004). These facts imply that, even with the task constraints mentioned, greater executive resources are not always recruited when imagining the future versus remembering the past.

Secondly, recent studies on autobiographical memory have found that traditional cue-word paradigms actually involve high proportions of spontaneously activated or directly retrieved memories (e.g., Harris, O’Connor & Sutton, 2015; Uzer & Brown, 2017; Uzer, Lee & Brown, 2012). This finding questions the basic assumptions of the prominent autobiographical memory model, which considers autobiographical memories as being predominantly constructed by top-down activation processes within the hierarchical autobiographical memory system (see Conway, 2005; Conway & Pleydell-Pearce, 2000). In a landmark replication of these studies with future imagined events, using the cue-word paradigm, Jeunehomme and D’Argembeau (2016) found that around 60% of future thoughts were reported by participants as being ‘directly accessed’ rather than effortfully constructed. Directly accessed thoughts were defined as future thoughts that came to mind rapidly and effortlessly under standard cue-word paradigm conditions.^1^ Across three experiments, such thoughts were indeed accessed more rapidly (with median response times varying between 3.0 and 4.4 s) than those classed by participants as generative thoughts (with median response times varying between 7.5 and 17.1 s), showing that they were associated with less effort. The existence of such a large percentage of subjectively ‘spontaneous’, directly-accessed thoughts in the context of a paradigm designed to elicit constructive processes is somewhat surprising and difficult to explain (cf. Jeunehomme & D’Argembeau, 2016).

In fact, this finding alone implies that other methods used to elicit voluntary future thoughts, may also involve spontaneous processes. For instance, it is possible that speeded tasks such as the future fluency tasks (MacLeod & Byrne, 1996), and sentence completion tasks (Anderson & Dewhurst, 2009) may involve a type of spontaneous chaining process (see Mace, 2009, for evidence of chaining in memory). Future studies will be needed to validate these claims. Nevertheless, just as has happened in memory research (Hintzman, 2011), future thinking researchers are now asked to question the assumption that voluntary processes are the norm.

Thirdly, the new area of spontaneous future cognition has emerged in recent years, defined as “unintended thought, related to the future, that comes to mind with little effort and little control over its content” (p. 635, Cole & Kvavilashvili, 2019). This field has established that spontaneous future thinking is commonly experienced in the laboratory and daily life, and is subjectively experienced with similar vividness and feelings of mental time travel as voluntary future thinking (Berntsen & Jacobsen, 2008; Cole, Staugaard & Berntsen, 2016). We note two important findings: (1) a large proportion of mind wandering experiences elicited in the laboratory (Baird, Smallwood & Schooler, 2011; Smallwood, Nind & O’Conner, 2009; Stawarczyk, Majerus, Maj, Van der Linden & D’Argembeau, 2011; Stawarczyk, Cassol & D’Argembeau, 2013) and daily life (Andrews-Hanna, Kaiser, Turner, Reineberg, Godinez et al., 2013; Song & Wang, 2012) are focussed on the future, rather than the present, or past (see Stawarczyk, 2018, for a review); (2) experiences of spontaneous future mental time travel in daily life and the laboratory are as common as involuntary memories, and are similar on several dimensions: they are often triggered by external cues, regularly refer to distinctive episodes and come to mind more rapidly than constructed events (see Berntsen & Jacobsen, 2008; Cole et al., 2016). The key point is that there is now converging evidence from both mind wandering and mental time travel research that spontaneous future thinking is common, perhaps more so than voluntary future thinking (e.g., Plimpton, Patel & Kvavilashvili, 2015; Seli, Risko, & Smilek, 2016; Seli, Maillet, Smilek, Oakman & Schacter, 2017; Stawarczyk et al., 2011; Warden, Plimpton & Kvavilashvili, 2019). The potential importance of this area is reflected in the rapid expansion of directly relevant studies examining its cognitive underpinnings, developmental trajectory and individual differences (see a special issue on Spontaneous Future Cognition in Psychological Research, 2019). Thus, a question emerges: If spontaneous future thinking is such a common aspect of daily life, why have experimental paradigms been used that restrict or prevent examination of this important type of future cognition?

## A dual process account of future thinking

In the present account of future thinking, we propose that in certain laboratory conditions (e.g., when completing monotonous and undemanding ongoing tasks) and especially in everyday contexts, future thinking can arise in an automatic way. This dual-process account complements and extends the currently dominant constructive process account and, we believe, provides more accurate fit for the diverse set of available data. For clarity, we do not imply that previous research on voluntary future thinking has been erroneous, or non-relevant. On the contrary, our dual process approach harnesses and adopts many insights from voluntary future thinking. However, here we aim to highlight and add the role of spontaneous cognitive processes to the investigation of future thinking by discussing its possible mechanisms, characteristics and functions.

At the outset, we need to clarify the terminology used to denote constructive and effortful processes in voluntary future thinking on the one hand, and the unintended nature of spontaneous future thoughts, on the other. This should also help to contextualise spontaneous future thinking, before we highlight its features below. One conceptual approach adopted in several studies using the descriptive experience sampling method (Hurlburt, 2011), assumes that rather than being distinct in kind, spontaneous future thoughts differ from ‘voluntary thoughts’ only by degree (i.e., they are characterised by less intention and less effort). Therefore, participants in these studies are asked to rate the intentionality of thoughts not as a dichotomy, but on a scale where only the end points correspond to the options ‘spontaneous’ and ‘deliberate’ (e.g., Martinon et al., 2019). However, in our view, this does not capture the qualities of spontaneous future thoughts, which become apparent when subtypes of future thinking are examined within a table, depicting types of future thinking in terms of intention and effort (see Table 1). While levels of effort involved in deliberately constructing a future scenario can vary along a continuum, the intention to construct such a scenario is either present or absent because one cannot intend to construct a future event only slightly or strongly. In other words, one is either in the ‘construction mode’ (similar to ‘retrieval mode’ used in the literature on episodic memory) or not. In line with this argument, several studies have used a dichotomous approach, where participants are asked to report if a thought was spontaneous or intentional (Barzykowski, Radel, Niedźwieńska & Kvavilashvili, 2019; Plimpton et al., 2015; Seli et al., 2017; Vannucci, Pelagatti, Chiorri & Brugger, 2019).^2^

**Table 1:** Two-dimensional structure of categorising future thoughts in terms of presence or absence of intention to have a future thought and levels of effort involved in imagining a future event

	Intention	
	Yes	No
Effort		
Yes (low to medium to high)	Generatively accessed future thought	?
No or minimal effort	Directly accessed future thought	Spontaneous future thought

“?” indicates that there is little empirical or theoretical support for this type of future thought

Within this context, spontaneous or involuntary future thoughts are clearly distinguishable from other subtypes of voluntary future thoughts elicited directly or generatively within the word-cue paradigm, by having no intention to imagine or construct a future thought, which phenomenologically translates into future thoughts popping into one’s mind unexpectedly and without effort (see Barzykowski & Staugaard, 2016, for a similar conceptualisation of involuntary autobiographical memory).^3^ This conceptual structure is also useful in highlighting the absence of future thoughts, which would fall into a cell designated by “?” in Table 1 (a perhaps implausible case where thinking about a future event is effortful but is not intended to take place).

In summary, this two-dimension structure appears helpful as it places known subtypes of future thinking into one of three cells and is contrary to the view that spontaneous future thoughts only differ from other subtypes by degree. Nevertheless, given that both spontaneously occurring and directly experienced voluntary future thoughts are characterised by a lack of (or minimal) effort and strategic construction processes, investigating similarities and differences between these two forms of future thinking is clearly an important avenue for future research (for similar research on involuntary and directly recalled autobiographical memories see Barzykowski & Staugaard, 2016; Barzykowskyi, Niedźwieńska & Mazzoni, 2019).

The guiding assumption of our dual-process view is that vivid episodic future thoughts can be elicited via two routes: via either a controlled or spontaneous fashion, and that the mode of elicitation is influenced by the characteristics and requirements of the situation (see Table 2). These situations/contexts are now quite widely documented, from laboratory studies that either instruct participants to wilfully generate future scenarios, or allow thoughts to flow freely (see Cole et al., 2016; Plimpton et al., 2015). To substantiate the claim that spontaneous future thinking is a genuine and substantial component of future thinking, we provide summaries of the cognitive mechanisms, characteristics and functions of spontaneous and voluntary future thinking (see Table 2, for a summary). We do not aim to summarise all relevant research, rather, we use exemplar studies from each domain to illustrate dual processes of future thinking.

**Table 2:** Differentiating processes, characteristics, paradigms and functions of voluntary and spontaneous future thinking

	Voluntary future thinking	Spontaneous future thinking
(a) Cognitive processes	Intentional/deliberate	Unintended/automatic
	Slow, effortful	Fast, effortless
	Self-directed	Undirected
(b) Characteristics of future thoughts	Semantic and episodic	Largely episodic
	Emotional and non-emotional in valence and emotional impact	Largely emotional in valence and impact
(c) Popular paradigms	Cue-word method	Vigilance task
(d) Possible functions	Simulating novel events/scenarios	Orienting to one’s existing current concerns, upcoming tasks and goals
	Creating and developing plans	Aiding the completion of pending goals and prospective memory tasks

(b) Evidence for differences in characteristics is derived from Berntsen and Jacobsen (2008), Finnbogadóttir and Berntsen (2011), Cole et al. (2016)

(c) evidence for differences in methods is derived from Addis et al. (2008), Cole et al. (2016), Hassabis et al. (2007) (see also Schacter 2012, for a review)

(d) Indirect evidence for differences in functions comes from: Anderson (2012), who showed that voluntary future thinking can integrate different sources of information in a flexible way, and Spreng et al. (2010) who demonstrated how this function could be harnessed when engaging in autobiographical planning. Evidence for creating novel scenarios or plans voluntarily, is derived from over a decade of research using instructions emphasising novelty of the to-be-constructed events in the cue word paradigm (e.g., Addis et al. 2008). Results from this research contrast the findings on spontaneous future thinking by Cole et al. (2016) who found higher ratings of novelty for voluntary compared with spontaneous future thinking (for similar points, see Jeunehomme and D’Argembeau 2016). In terms of the functions of spontaneous future thought, we draw upon research showing a link between spontaneous future thought sand current concerns (e.g., Cole and Berntsen 2016) and prospective memory (e.g., Kvavilashvili and Fisher 2007; Szarras and Niedźwieńska 2011; for a review, see Kvavilashvili and Rummel 2019)

### Voluntary future thinking

The deliberative, slow and self-directed cognitive processes involved in constructing future events is well-specified (e.g., Hassabis & Maguire, 2007; Schacter & Addis, 2007; Schacter, 2012, see Table 2). As these processes have been reviewed elsewhere (Schacter, 2012; Schacter et al., 2017)—here we simply lay out the essential elements of the hypothesised processes involved in voluntary episodic future thinking.

First, we draw upon over a decade of empirical research that attempted to delineate the cognitive processes underlying voluntary future thinking. We specify two main phases of this cognitive process, first outlined by Addis et al. (2007). The first has been termed the construction phase and represents the ability to wilfully and consciously use the cue word task parameters to generate a future event by selecting the most appropriate details from memory (i.e., people, places, objects; see Addis, Musicaro, Pan & Schacter, 2010). Although generative memory retrieval is seen as constructive (and prone to error, Schacter, 2012), the process is aided by having a single temporal context to which details originally belong (see Anderson et al., 2012 for similar arguments). In future event construction, the flexible and complex nature of identifying and then selecting appropriate details from long-term memory to combine them into a novel ‘whole’ is presumably a key factor explaining why a link between executive processes and future thinking performance has been found in some studies, especially in the construction phase (Addis et al., 2007; Anderson et al., 2012; D’Argembeau, Stawarczyk, Majerus, Collette, Van der Linden et al., 2010; de Vito et al., 2012). These cognitive processes would naturally recruit a broad set of processes collectively labelled executive function (see Miyake, Friedman, Emerson, Witzki, Howerter et al., 2000; Stuss & Alexander, 2002).

The second phase has been termed the elaboration phase, and represents the vivid unfolding of the scene in one’s mind, after a scene is constructed (Addis et al., 2007). As such, areas associated with visual imagery become active here, as do those linked with self-referential processing (Addis et al., 2007; see also D’Argembeau et al., 2010a). However, elaboration is assumed to involve subjective ‘pre-experiencing’, and not necessarily executive function (as shown by comparisons with the elaboration phase in future thinking and episodic remembering, Addis et al., 2007). Thus, we hypothesise that the processes of elaboration will be similar in voluntary and spontaneous future thought. We therefore see construction as the defining feature of voluntary future thinking, in agreement with several authors (Addis et al., 2007; D’Argembeau et al., 2010a; Schacter et al., 2012).

Episodic future construction can also be seen as the encoding of a new memory (of an imagined future event), and rather than encoding a lived experience, as would happen in autobiographical memory (Conway, 2005), an ‘event’ or scenario is encoded as a mental representation. Neuroscientific research has carefully disentangled the encoding components of episodic future thinking, using a subsequent memory paradigm to establish the neural correlates of successful ‘event’ encoding (Martin, Schacter, Corballis & Addis, 2011). This process, in our view especially associated with voluntary future thinking, may hold important functions.^4^ In particular, the ability to “foresee, plan, and shape virtually any specific future event” (p. 299, Suddendorf & Corballis, 2007) has been proposed as a key function of episodic future thinking, from those emphasising either evolutionary or everyday functions (Schacter, 2012; Schacter et al., 2017; Suddendorf & Corballis, 2007; Szpunar, 2010).

We hold that the ability to create novel scenarios in an iterative fashion, in which increasingly effective plans are created (Suddendorf & Corballis, 2007), garners wide-ranging benefits, not only to personal, but also societal planning (Szpunar & Szpunar, 2016, see Jeunehomme & D’Argembeau, 2016 for a related point). Humans can create hoped-for and feared possible selves (Markus & Nurius, 1986), and devise plans in relation to these (Murru & Martin Ginis, 2010). In applied psychology, although ‘if–then’ plans within research on implementation intentions have proved effective (Gollwitzer, 1999), episodic future thinking has been shown to improve those plans (Knäuper, McCollam, Rosen-Brown, Lacaille, Kelso & Roseman, 2011), perhaps by stabilising the memory trace of the future simulation. Similar findings have been found in prospective memory, where the manipulation of episodic future simulation has been shown to ‘boost’ the encoding of prospective memories (e.g., Altgassen et al., 2015; Neroni, Gamboz & Brandimonte, 2014).

Of course, other ways in which voluntary future thinking could be harnessed must be acknowledged (see Szpunar, Spreng, & Schacter, 2014). To name a few, humans might use the ability to construct novel and coherent episodic future thoughts for encoding lists (Klein, Robertson & Delton, 2010; Grilli & Glisky, 2011), intentional mental practicing of tasks (Driskell et al., 1994; Suddendorf, Brinums, & Imuta, 2016), increasing empathic behaviour (Gaesser & Schacter, 2014) or reducing impulsiveness (Daniel, Stanton & Einstein, 2013). In short, voluntary future thinking allows humans to creatively construct novel scenarios and envisage plans. These main functions are highlighted in Table 2.

Where we differ from previous approaches is by indicating that voluntary future thinking is not the dominant or the only type of future thinking in one’s cognitive arsenal. Specifically, in our view, with precise objectives (i.e., to create an original plan), one can have flexibility and control over what one imagines, thus taking advantage of the various benefits of the voluntary mode. Nevertheless, evidence abound that future thinking does not rely on a unitary process (see Tables 1 and 2), and where construction processes are not involved (see below for examples of functions of spontaneous future thinking), some, or perhaps all, of these functions may not apply.

### Spontaneous future thinking

Drawing upon recent work, spontaneous future thinking is characterised here as automatic, fast and undirected (see Table 2) (Berntsen & Jacobsen, 2008; Finnbogadottir & Berntsen, 2011; Cole et al., 2016; for a definition, see Berntsen, 2019; Cole & Kvavilashvili, 2019). As stated at the beginning of this article, the key question in future thinking research concerns the paradox of having fully-fledged episodic future thoughts in the absence of wilful constructive processes. Fox and Christoff (2018) elaborated this idea by stating “the degree to which mental processes that are ostensibly spontaneous and beyond our control appear to be planned, relevant, and insightful with respect to our personal goals and concerns is striking” (p. 5). Within this quote they have also hinted at a solution and our central hypothesis concerning spontaneous future thoughts. We propose that spontaneous future thoughts are best characterised as instances of ‘pre-made’ future thoughts or ‘memories of the future’ returning to consciousness after once being constructed in the past (see Jeunehomme & D’Argembeau, 2016; Ingvar, 1985; Szpunar, Addis, McLelland & Schacter, 2013). We believe that the pre-made hypothesis offers a solution to the paradox, as well as why spontaneous future thoughts are prevalent, occur with fluency and why they predominantly refer to temporally close and goal-related happenings.

We believe several hypotheses about spontaneous future thinking can be discarded from the outset. It is clear from several studies (e.g., Cole & Berntsen, 2016; Cole et al., 2016; Mazzoni, 2019; Plimpton et al., 2015; Warden et al., 2019) that they are not ‘fantasy-laden’ and ‘random’, or re-interpretations of past memories as future-oriented thoughts (if the latter were true, experimenter and participant ratings into temporal categories of past, present, and future would be incongruent, but this is not the case, Plimpton et al., 2015; Barzykowski et al., 2019; see Cole et al., 2016 for similar arguments).

Rather, spontaneous future thoughts are most often about concrete, upcoming events and/or planned tasks and goals, which have been previously constructed and/or deliberately thought of, and pop into mind in the delay interval before the event actually has taken place. Evidence for this can be found across a range of recent studies on spontaneous future thinking. The most important and direct evidence comes from those laboratory and naturalistic experience-sampling studies that have specifically examined the content of participants’ spontaneous future thoughts (for a review of these studies, see Kvavilashvili & Rummel, 2019). For example, using a laboratory vigilance task with probes in which participants had to describe their thoughts at that moment, Plimpton et al. (2015) showed that plans and intended actions (e.g., “I remembered that I need to book some days out with friends and for myself”, “I must text X for a dinner date”) made up a large percentage (60%) of spontaneous future thoughts. A further 38% of spontaneous future thoughts referred to scheduled events in near future without specifying a particular intention (e.g., “thinking about my upcoming holiday to Cork”, “job interview I have next week”). Using a different version of the vigilance task, Mazzoni (2019) replicated and extended these findings by showing that spontaneous future thoughts were significantly more likely to be plans rather than imagined scenes of future events, and that thinking about planned actions involved less cognitive resources than thinking about future events (as these thoughts did not reduce in number during a more cognitively demanding vigilance task). It is important that the prevalence of thoughts about future plans has also been reported in several naturalistic diary and experience sampling studies (e.g., Anderson & McDaniel., 2019, Study 1; Baumeister, 2018; D’Argembeau, Renaud & Van der Linden, 2011; Warden et al., 2019).

Importantly, these studies have also shown that not all spontaneous future thoughts were ‘pre-made’, because occasionally participants reported experiencing novel spontaneous thoughts about the possible future plans or events that they had not constructed before. In other words, such minority cases point to an interesting possibility that sometimes novel constructions can come to mind automatically without strategic effortful processes (see also Jeunehomme & D’Argembeau, 2016, for the discussion of this point in relation to directly accessed future thoughts within the cue word paradigm). Further evidence for such a possibility comes from recent studies by Szpunar et al., in which participants were asked to report spontaneous thoughts about the imminent future (i.e., future scenarios that could happen in the next few seconds or minutes, in the context of the current situation, e.g., Puig & Szpunar, 2019). Puig and Szpunar’s preliminary evidence indicates that such mental representations have unexpected (i.e., novel) content, are largely negative and may serve behavioural functions (e.g., if imagining a car crash while driving, one might drive considerably slower). Such immediate spontaneous future thoughts may be a highly functional evolutionary precursor to the voluntary future thoughts that enable humans to achieve goals in future contexts (see Suddendorf & Corballis, 2007 for more elaborated and related evolutionary arguments).

Further evidence for the ‘pre-made’ hypothesis of spontaneous future thoughts and why they come to mind rapidly (Cole et al., 2016), comes from studies that have compared the temporal distribution of thoughts. Thus, compared to voluntary future thoughts, spontaneous future thoughts are often about temporally near events referring to tasks and events occurring later in the same day or in the next few days (for a review, see Kvavilashvili & Rummel, 2019; see also Berntsen, 2019, for a re-analysis of data from three studies). Presumably, concrete and previously constructed plans would likely refer to temporally near happenings, and this accords with data from several studies (Berntsen & Jacobsen, 2008; Cole et al., 2016; Plimpton et al., 2015).

Yet another way of assessing the ‘pre-made’ hypothesis is to ask participants to introspect about the frequency and the source of their spontaneous future thought content. For example, using the vigilance task and the standard cue word paradigm to assess spontaneous and deliberate thoughts about the future and the past, respectively, Cole et al. (2016) found that ratings of rehearsal (How often have you previously thought about the imagined future event?) made on a 5-point scale (1, never; 5, very often) were the highest for spontaneous future thoughts when compared to all other types of thought, which did not differ from each other. In another (unpublished) study, using the same vigilance task, participants were asked the extent to which their future thoughts contained ‘exactly the same’ configuration of details as a previously constructed future thought (Cole, Barnes, Jones, & Elwell, 2018). Of all spontaneous future thoughts, the most frequently provided response indicated that participants experienced almost ‘exactly the same’ (4 on a scale of 1–5, 5 being ‘exactly the same’) content as a previously-constructed future event. Voluntary future thoughts, on the other hand, were significantly less likely to be reiterations of previously constructed thoughts, and neither spontaneous nor voluntary future thoughts were defined by participants simply as memories recast or reinterpreted as future-oriented (such as a dentist appointment from the past re-interpreted as a representation of the future). In other words, voluntary future thoughts were not simply replays of the past, and fulfilled the typical definition of a constructed novel event, and spontaneous future thoughts were largely based on the content of previous future event constructions.

Broadly similar findings were obtained also by Jeunehomme and D’Argembeau (2016) on directly accessed future thoughts elicited in the context of the cue word paradigm to study voluntary future thinking (but instructing participants to think of plausible future events without emphasising the need to produce novel events). Across three experiments, their results showed that the vast majority of directly accessed thoughts, which came to mind ‘fully-formed’ as specific episodic scenarios, had been thought of previously (i.e., they were not novel) and the frequency of previous construction (rated on a scale by participants) predicted the likelihood of a direct versus generative process. In addition, the likelihood of direct response was significantly increased by perceived probability that the imagined event was going to actually happen in the future (Exp. 1). Based on these findings, Jeunehomme and D’Argembeau (2016) concluded that most directly accessed episodic future thoughts could be conceptualised as ‘memories of the future’ rather than newly constructed or imagined future events, and highlighted the potential importance of such pre-stored mental representations of the future in successfully managing goal-directed and planned behaviours in everyday life (see also Baumeister, Maranges, & Sjåstad, 2018; Baumeister, Oettingen & Vohs, 2016).

If one accepts the premise that spontaneous future thoughts are primarily pre-made constructions, and ‘memories of the future’, it would necessarily follow that they would come to mind with little effort and no intent (the defining features of spontaneous future thoughts, Cole & Kvavilashvili, 2019, see Tables 1 and 2). It also becomes clear how they can be re-instated in consciousness with such rapidity when semantically-related external cues appear in the environment (Berntsen & Jacobsen, 2008; Cole et al., 2016; Plimpton et al., 2015), or even when deliberate construction is attempted (Jeunehomme & D’Argembeau, 2016). Commonalities between involuntary memories and spontaneous future thoughts (e.g., in specificity, vividness, latency; Cole et al., 2016) also become explicable, when one assumes both rely on the reactivation of a memory.

Furthermore, if we assume that spontaneous future thoughts are often pre-made, and can be cued not only by external stimuli, but also by internal thoughts (see Warden et al., 2019), it is understandable why mind wandering studies that do not use meaningful external stimuli find high rates of future thoughts (Baird et al., 2011; Smallwood et al., 2009). It also explains why prospective memory, mental time travel and mind wandering studies, in which participants record their everyday thoughts, find that future-oriented spontaneous thoughts are highly prevalent, as they are not only ‘memories of the future’, but are also goal-relevant and sensitised to the many potential cues experienced in daily life (D’Argembeau et al., 2011; Kvavilashvili & Fisher, 2007; Warden et al., 2019). In other words, they represent previously formulated goals and intentions to be completed at some point in the future (for more detailed discussion, see Kvavilashvili & Rummel, 2019).

We believe that this hypothesised cognitive process holds many functional benefits. First, within our account, spontaneous future thoughts would fulfil the criteria of a goal ‘reminder mechanism’ (Klinger, 2013). Second, it would serve to remind people of prospective memory tasks they need to carry out in the future (Kvavilashvili & Fisher, 2007; Szarras & Niedźwieńska, 2011; see Mazzoni, 2019, for related arguments). Third, it may strengthen the intention-superiority effect in prospective memory, which refers to intention related contents having heightened levels of activation compared to other contents stored in memory (Goschke & Kuhl, 1993). If individuals may ‘pre-experience’ intended acts spontaneously in the delay, this would further strengthen the representations of those acts in one’s memory. Finally, it may fit into the prescient model of mental time travel and decision-taking put forward by Boyer (2008), in which involuntary or spontaneous remindings served to reduce impulsive behaviour.

In short, spontaneous future thinking may garner far more benefits than we currently acknowledge. Take this example. An office worker constructs a mental image of handing their colleague a birthday card when they arrive at work. However, typically there would be no explicit reminders to help the worker to deliver the letter when at work—the time when a goal-oriented response is needed. Hence, a self-reliant system that regularly re-activates pre-made plans, and is sensitised to be triggered internally or by external cues, is ideally suited to re-orient the individual to their goal and ensure the card is handed over. In short, such a process is highly adaptive and it is no surprise that it is prevalent in daily life (e.g., Ellis & Nimmo-Smith, 1993; Sellen, Louie, Harris & Wilkins, 1997; Szarras & Niedźwieńska, 2011).

The links between spontaneous future thinking and goal-oriented cognition notwithstanding, we note here that we do not perceive the functions identified in Table 2 as the only ones related to spontaneous future thinking. Indeed, it is plausible that spontaneous future thinking fulfils other functions, some of which have been indicated in the mind wandering literature (e.g., emotion regulation, see Ruby, Smallwood, Engen & Singer, 2013; see also Smallwood & Schooler, 2015). We call on other researchers to explore different functions, and specifically compare functions of voluntary and spontaneous future thought in single studies. We note the current dearth of such research (although see Duffy & Cole, 2019). But in this position paper, our focus remains on the relevance of spontaneous future thinking to goal-oriented cognition and behaviour, which coheres with several well-established theoretical accounts (e.g., Klinger, 2013, see below).

### Relation to other theoretical approaches

Theoretical progress has already been made by Berntsen et al. , who proposed that spontaneous future thinking (and involuntary memory) is an evolutionarily earlier function than voluntary (past and) future thinking (Berntsen, 2012; Berntsen & Jacobsen, 2008). They also proposed that the former had unique benefits by providing an ongoing and effort-free way that “helps us to maintain a wider time horizon with low cognitive costs” (p. 304, Berntsen, 2012).

A potential cognitive mechanism of spontaneous future thinking was also proposed by Berntsen and colleagues by suggesting that the occurrence of involuntary thoughts about the future, much like involuntary autobiographical memories “would owe their existence to spreading activation in complex associative networks for autobiographical information” (p. 1102, Berntsen & Jacobsen, 2008). In a more recent paper, Berntsen (2019) discusses similarities between the temporal distribution curves for past and future events and questions whether this could reflect the exact same mechanisms underlying spontaneous mental time travel about the past and the future. However, according to Berntsen (2019) such an “explanation would force us to radically rethink theories of forgetting because frequently invoked forgetting mechanisms (e.g., interference and decay) operate on already encoded and stored information. Such explanations would not work for imagined future events since future events have not yet been encountered and encoded” (p. 656). We take a different stance from Berntsen (2019) on the underlying mechanisms of spontaneous future thoughts. Under the pre-made hypothesis, such forgetting processes would clearly operate on spontaneous future thoughts, as they rely on well-known memory processes, following their known and well-defined temporal trajectory (see Szpunar et al., 2013, for a review of the ‘memories of the future’ hypothesis concerning voluntary future thinking). This dual-process account therefore solves the question of similarities between the temporal distribution of spontaneous past and future thoughts.

Our explanation of the process that underlies spontaneous future thoughts is most consistent with Klinger’s current concern theory (Klinger, 1975, 2009). The theory of current concerns indicates that, at any one time, humans have a highly active set of goals that they are committed to but have not yet been completed or discarded. These goals are sensitive to cues emanating from the external environment or internal train of thought, wholly or partially related to that goal. Findings from spontaneous future thoughts not only indicate that they are highly goal-related (e.g., Cole & Berntsen, 2016), but also that they are activated by cues, especially in the external environment (Cole et al., 2016; Plimpton et al., 2015; Warden et al., 2019).

This dual process account of episodic future thinking also fits nicely with the recently proposed extension of the autobiographical Self Memory System by Conway et al. (2019), which incorporates into the autobiographical memory system voluntary episodic future thinking on the one hand (described above), and what has been termed the remembering-imagining system (RIS; Conway, Loveday and Cole, 2016), on the other.

The RIS operates within the present time-frame of this extended autobiographical self-memory model. According to Conway et al. (2016), “there is what we conceive of as an extended form of consciousness that consists of memories of the recent past and images and expectations of the near future, and it is this form of extended consciousness that we have termed the RIS.” (p. 257). This increased awareness of temporally-near past and future episodes specifies an extended present time-frame—an extended ‘now’. Data presented by Conway et al. (2016), together with findings from Spreng and Levine (2006) and Berntsen’s laboratory (see Berntsen, 2019) support this hypothesis, at least for past and future thinking which involve constructive processes.

How does this fit within the proposed dual process account of future thinking? In Conway et al. (2016), the RIS was integrated into the Self Memory System (see Conway & Pleydell-Pearce, 2000; Conway et al., 2019). In so doing, past and future thinking was seen in the context of a goal-oriented cognitive system whereby abstract goals (e.g., becoming healthy), are fed into lifetime periods (e.g., training for a 10 km race) leading to the construction of temporally-near episodic future thoughts (e.g., running 5 km in the park tomorrow, see Conway et al., 2019). The link between goals and constructed episodic future thoughts is well-evidenced (D’Argembeau & Mathy, 2011; D’Argembeau et al., 2011; Spreng & Levine, 2013). Critically, when carried out, what were episodic future thoughts become a set of highly accessible temporally-near goal-related episodic memories (Conway et al., 2019). Thus, a goal-oriented cognitive system is delineated.

In our view, spontaneous past and future thoughts naturally emanate from the RIS and this extended autobiographical memory system. Specifically, if constructed episodic future thoughts often represent to-be-completed tasks that are themselves related to abstract goals, it is unsurprising that future thoughts recorded in mind wandering, prospective memory and involuntary future thinking studies are mostly related to temporally-near planned tasks and upcoming events (e.g., Plimpton et al., 2015; Warden et al., 2019, see above sections for more empirical support). Functionally, these ‘pre-made’ representations of upcoming future events and planned actions, which reside at the bottom layer of the hierarchy in the form of ‘episodic details’ or memories, would then be sensitised to external and internal cues, aiding their behavioural completion (see Klinger, 1975; Kvavilashvili & Rummel, 2019; see also Jeunehomme & D’Argembeau, 2016 for a similar proposal regarding directly accessed future thoughts).

Developing these ideas further, under the updated self memory system (Conway et al., 2019), episodic future thoughts are constructed via activation through levels of autobiographical knowledge (see Fig. 1). While we agree that voluntary future thinking may operate in this way, the dual process approach outlined here differs by assuming different cognitive processes for spontaneous future thinking. Specifically, Conway et al. (2019) propose that event-specific details (e.g., people, places objects) from the bottom layer of autobiographical knowledge are utilised and integrated into episodic future thoughts, incorporating details which are essentially ‘atemporal’ (until they are linked with coherent autobiographical memories or future thoughts). However, in our framework, upcoming events and plans have already undergone detail integration, and thus exist as an integrated ‘whole’ (consisting of a set of bound episodic details), that can be accessed and brought to mind spontaneously.^5^ Although such pre-made representations are not included in Conway and colleagues’ model, considering they are highly specific, placed within this model, they would logically form a subset of representations in the lowest level of autobiographical knowledge (but would be combined with personal semantic information—i.e., contextualised within lifetime periods and self-images—when brought to consciousness).^6^

Although the RIS and Conway’s updated autobiographical memory system is still primarily focussed on explaining voluntary and constructive processes in past and future mental time travel, we believe that they represent useful theoretical frameworks to understand the existence and processes underlying spontaneous future thought, leading to testable research questions for researchers working on future thinking or related areas.

## Future directions

The approach to future thinking, described in this article, leads to several open-ended questions and avenues for future investigations. Here, we specify what we see as the main questions in the field. It is clear from emerging studies focussing on spontaneous future thinking, that a natural and helpful strategy has been to compare this phenomenon with related areas such as involuntary memory and voluntary future thinking. How, then, can we test the dual process account using such comparisons?

Findings based on introspection may be particularly informative. As described in the study above (Cole et al., 2018), researchers can ask participants whether a particular spontaneous future thought has been constructed before. If the pre-made hypothesis is correct, one would expect participants to rate the event high on a scale of previous construction (see Cole et al., 2016; Jeneuhomme & D’Argembeau, 2016, for initial evidence). In the case of voluntary future thoughts, a wealth of research has demonstrated that these can, and often are, novel at the time of construction, thus participants should identify these as novel (Addis et al., 2007, 2010; D’Argembeau & Van der Linden, 2004). Indeed, the novelty imbued by constructive processes may be a function unique to voluntary future thinking (see Table 2). Overall then, differences should be found in the extent to which spontaneous and voluntary future thoughts are previously constructed. Of course, these findings are only valid to the extent that an individual can accurately recall whether a spontaneous future thought has been constructed, and research will be needed to clarify how such introspective judgments are made.

Contrasting spontaneous future thoughts with involuntary autobiographical memories on measures of introspection may be another interesting avenue for investigation. Specifically, participants may indicate that spontaneous future thoughts had been previously thought about more frequently than spontaneous thoughts about the past (cf. covert rehearsal, Johnson, Foley, Suengas, Raye, 1998). Another question is whether ‘rehearsal’ is positively correlated with the frequency of occurrence of such spontaneous thoughts (cf. Jeunehomme & D’Argembeau, 2016, for a similar finding in the context of direct access). In addition to introspection, as both are encoded, researchers may find it useful to examine forgetting curves of the original experience (in the case of involuntary autobiographical memories) or imagined ‘event’ (in spontaneous future thought). Future research will be needed though to distinguish the contribution of basic forgetting processes versus a possible general preference to report more upcoming future thoughts.^7^ Researchers may also adopt experimental methods (e.g., Mazzoni, 2019; Vannucci et al., 2019) to examine how voluntary constructions affect subsequent past and future spontaneous thoughts.

Another interesting question links back to the RIS (Conway et al., 2016) and the intention superiority effect in prospective memory research and whether the representations of upcoming future plans and events accrue higher levels of activation than representations of past events. Some initial evidence for this idea comes from the results of mind-wandering studies with the Sustained Attention to Response Task where a prospective bias is predominantly found when participants are not exposed to meaningful irrelevant or incidental cues (e.g., Baird et al., 2011, see also Warden et al., 2019; when verbal cues are present people tend to think more about the past, as shown by Vannucci et al., 2017). The central question is that if a future thought arrives in consciousness without any noticeable external or internal cue, this thought should be more highly activated than the representation of the thought that is triggered by a cue. Future research is needed to tackle this issue further.

Other empirical questions arise from exploring differences between different subtypes of spontaneous future thought, such as specific intentions or plans to do something (e.g., buying a present for a friend’s birthday) and upcoming events that do not specify any intended actions in relation to them (e.g., a job interview next week) (as reported by Plimpton et al., 2015 and Warden et al., 2019). Although upcoming intentions and events may be previously constructed and related to higher-order goals such as ‘being a good friend’ and ‘finding a satisfying job’, respectively (see Cole & Berntsen, 2016), spontaneous future thoughts representing plans and prospective memory tasks may serve to initiate goal-oriented thoughts and behaviour (Klinger, 1975; Klinger, Marchetti, & Koster, 2018), while spontaneous thoughts about upcoming events may serve the function of rehearsing the contents or components of upcoming events (e.g., types of questions that may come up during the interview). For example, when Stawarczyk, Majerus and D’Argembeau (2013) falsely informed their participants that they would perform either a stressful task (a videotaped speech about one’s physical appearance) or a neutral task (a simple visual planning task) following the sustained attention to response task with thought-probes, they found that more than 25% of reported mind-wandering episodes in the experimental group were described as attempts to prepare for the subsequent task versus only 2% in the control group (see also Steindorf & Rummel, 2017). More specific investigations into differences between the functions served by spontaneous future thoughts about upcoming plans/intentions and events will be necessary to address this question. A related line of research should investigate in more detail the frequency and nature of those spontaneous future thoughts about hypothetical events and scenarios, which appeared to be novel constructions (i.e., did not seem to be “pre-made”) as reported in several studies (Plimpton et al., 2015; Puig & Szpunar, 2019; Warden et al., 2019), to examine an interesting theoretical question whether completely novel events can come to mind without intention and effortful deliberation and construction processes.

Finally, although understanding of voluntary and involuntary or spontaneous future thought has increased in recent years, the dynamic interplay between these modes of future thinking is poorly understood. In short, what is the interplay between spontaneous and voluntary future thinking in cognitive tasks and daily life? Even if most future thoughts are triggered spontaneously, are controlled cognitive processes required to ‘verify’ or ‘monitor’ these thoughts at later stages of processing? Some research has begun to tackle this question in the context of mind wandering (e.g., Pelagatti, Binder & Vannucci, 2018; Christoff et al., 2016; see Smallwood, 2013 for a review), showing that specific examination of the temporal dynamics of future thought is certainly a tractable avenue of research.

## Summary

One need only look at other areas of cognitive psychology to find useful dual-process frameworks, which have added substantial explanatory value (see Evans, 2008 for a review; Kahneman, 2011; McDaniel, Umanath, Einstein, & Waldum, 2015; Yonelinas, 1999). In our view, future thinking can be characterised by two processes that complement each other: Voluntary future thinking is required to construct or encode new ‘events’, and spontaneous future thinking is able to retrieve these future thoughts in an automatic fashion. The role of future thinking in goal-oriented cognition and behaviour has been clearly stated in many dominant theoretical accounts of future thinking and the self (Conway et al., 2019; Suddendorf & Corballis, 2007; Schacter & Addis, 2007). However, in addition to standard approaches, this dual process account explains how future thinking can aid future-oriented cognition and behaviour in a relatively effortless manner (and supports findings from prospective memory, mind wandering and mental time travel research). Specifically, a system is delineated whereby humans are periodically and vividly reminded of their currently active goals, making goal completion more probable (cf. Kvavilashvili & Rummel, 2019). However, we highlight the fact that more empirical work is needed to lend further support to this approach and evaluate alternative explanations (e.g., Berntsen, 2019).

Fundamentally, if future thinking were functionally important, it would not be efficient to use effortful future thinking when memory processes allow us to re-instate thoughts spontaneously. So, even though we have known of the existence of spontaneous future thinking for a decade (Berntsen & Jacobsen, 2008), the field may have neglected a tantalising possibility: That spontaneous future thinking is the default mode of imagining the future.

## Footnotes

1 Directly accessed future thoughts are similar in many respects to our definition of spontaneous future thinking. They arise swiftly and with minimal or no effort. However, they are differentiated from spontaneous future thoughts by arising in the context of being in the voluntary imagining mode, similar to being in the retrieval mode during standard autobiographical memory studies (e.g., see Barzykowsky & Staugaard, 2016).

2 It is interesting that even in studies that have used a continuous scale for rating intentionality of thoughts, for the purpose of analyses, thoughts have been sometimes dichotomised by labelling all the thoughts rated at the lower end of the scale as ‘spontaneous’ and at higher end of the scale as ‘deliberate’ (Jordão, Pinho, & St. Jacques., 2019).

3 This does not preclude the possibility that effort is required or necessary after the spontaneous future thought arrives in consciousness, which is aligned with the difference between occurrence and maintenance of mind wandering episodes (Smallwood, 2013).

4 This does not exclude the possibility that spontaneous future thoughts are also re(encoded) when brought to mind, and this may be relevant to how spontaneous future thinking aids task completion in prospective memory tasks. We thank an anonymous reviewer for raising this point.

5 Note that a pre-made future thought would cease to be ‘atemporal’ like other event-specific details on the lowest level of autobiographical knowledge, and would be instead treated as ‘future-oriented’ as it pertains to an upcoming event or future plan (i.e., it would have a temporal ‘tag’). Although event representations may be similar in content whether past- or future- oriented, we highlight that humans are able to recognise the temporal directions of internal representations, at least in healthy populations. Thus, past and future representations are typically not confused, perhaps due to their contrasting phenomenological characteristics (see D’Argembeau & Van der Linden, 2004; Johnson & Sherman, 1990 for relevant discussions).

6 We thank a reviewer for bringing this possibility to our attention.

7 We thank a reviewer for raising this point.
